# Inhibition of CK2/ING4 Pathway Facilitates Non‐Small Cell Lung Cancer Immunotherapy

**DOI:** 10.1002/advs.202304068

**Published:** 2023-10-23

**Authors:** Qian Gou, Huiqing Chen, Mingjun Chen, Juanjuan Shi, Jianhua Jin, Qian Liu, Yongzhong Hou

**Affiliations:** ^1^ Department of Oncology, the Affiliated Wujin Hospital of Jiangsu University Changzhou Jiangsu 213017 P. R. China; ^2^ School of Life Science Jiangsu University Zhenjiang Jiangsu 212013 P. R. China; ^3^ School of medicine Jiangsu University Zhenjiang Jiangsu 212013 P. R. China; ^4^ Changzhou Key Laboratory of Molecular Diagnostics and Precision Cancer Medicine of Wujin People's Hospital (the Wujin Clinical College of Xuzhou Medical University) changzhou Jiangsu 213017 P. R. China

**Keywords:** CK2, ING4, NSCLC, PD‐1/PD‐L1, tumor immune escape

## Abstract

Immune cells can protect against tumor progression by killing cancer cells, while aberrant expression of the immune checkpoint protein PD‐L1 (programmed death ligand 1) in cancer cells facilitates tumor immune escape and inhibits anti‐tumor immunotherapy. As a serine/threonine kinase, CK2 (casein kinase 2) regulates tumor progression by multiple pathways, while it is still unclear the effect of CK2 on tumor immune escape. Here it is found that ING4 induced PD‐L1 autophagic degradation and inhibites non‐small cell lung cancer (NSCLC) immune escape by increasing T cell activity. However, clinical analysis suggests that high expression of CK2 correlates with low ING4 protein level in NSCLC. Further analysis shows that CK2 induce ING4‐S150 phosphorylation leading to ING4 ubiquitination and degradation by JFK ubiquitin ligase. In contrast, CK2 gene knockout increases ING4 protein stability and T cell activity, subsequently, inhibites NSCLC immune escape. Furthermore, the combined CK2 inhibitor with PD‐1 antibody effectively enhances antitumor immunotherapy. These findings provide a novel strategy for cancer immunotherapy.

## Graphical abstract

ING4 acts as an autophagy receptor to induce PD‐L1 autophagic degradation leading to inhibition of tumor immune escape, whereas CK2 can induce ING4‐S150 phosphorylation and degradation by JFK ubiquitin ligase resulting in tumor immune escape. Importantly, CK2 inhibitor can effectively enhance PD‐1 antibody anti‐tumor immunotherapy by inhibiting CK2‐ING4 pathway. This study reveals a novel mechanism of the coordination of CK2 with ING4 to regulate PD‐L1 expression.

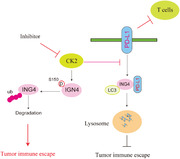

## 1 Introduction

ING4 (inhibitor of growth 4) is one of the members of inhibitor of growth (ING1‐5) family.^[^ ^1^ ^]^ As a tumor suppressor, ING4 negatively regulates tumor growth by inhibiting cancer cell proliferation, migration, and invasion,^[^ ^1^, ^2^ ^]^ while the loss of ING4 has been observed in multiple types of cancer including lung cancer,^[^ ^3^ ^]^ hepatocellular carcinoma (HCC),^[^ ^4^ ^]^ astrocytomas,^[^ ^5^ ^]^ ovarian,^[^ ^6^ ^]^ colorectal adenocarcinoma,^[^ ^7^ ^]^ and breast cacner.^[^ ^8^ ^]^ However, the effect of ING4 on tumor immune escape is still unclear. Although activation of the host immune response could kill cancer cells,^[^ ^9^ ^]^ and aberrant expression of some immune checkpoint proteins such as PD‐L1 or CD47 facilitates tumor immune escape.^[^ ^10^ ^]^ PD‐L1 is a “don't find me” signal that binds to PD‐1 on T cells resulting in the inhibition of T cell killing of cancer cells.^[^ ^11^, ^12^ ^]^ PD‐L1 gene expression is increased by aberrant activation of c‐Myc, Bromodomain‐containing protein 4 (BRD4), hypoxia response element (HRE), and signal transducer and activator of transcription 3 (STAT3).^[^ ^13^ ^]^ On the other hand, PD‐L1 protein level is regulated by proteasomal and lysosomal‐dependent degradation, which in turn enhances antitumor immunotherapy.^[^ ^14^ ^]^ Blockade of PD‐1/PD‐L1 immune checkpoint using antibodies could be an effective therapy for several types of cancer including melanoma, gastric cancer, breast cancer, and non‐small‐cell lung cancer (NSCLC),^[^ ^15^ ^]^ despite having low response rates (< 40%) for patients with unclear mechanism.^[^ ^16^ ^]^

As a constitutively active serine/threonine kinase, CK2(casein kinase 2) is a tetramer, which consists of two catalytic subunits (CK2α or CK2α’) and two regulatory subunits (CK2β). CK2α or CK2α’ is encoded by *CSNK2A1* or *CSNK2A2* gene, and CK2β is encoded by *CSNK2B* gene. CK2α/CK2α or CK2α/CK2α’ together with two CK2β forms a tetramer that exhibits catalytic activity for targeted substrates.^[^ ^17^ ^]^ Hundreds of substrates are phosphorylated by CK2 including Stat3, p53, Janus kinase 2 (JAK2), PTEN, RelA, and AKT,^[^ ^18^, ^19^ ^]^ leading to regulation of diabetes, cardiovascular diseases, angiogenesis, and tumor progression.^[^ ^20^ ^]^ CK2 is highly expressed in multiple types of cancer that regulates cancer cell proliferation, apoptosis, invasion, migration, and tumor progression,^[^ ^19^ ^]^ while the interaction of CK2 with ING4 on tumor immune escape is still unclear. Here we found that ING4 inhibited NSCLC immune escape by inducing PD‐L1 autophagic degradation, whereas CK2 reversed this event by promoting ING4 phosphorylation and degradation.

## 2 Results

### 2.1 Loss of ING4 Promoted Tumor Immune Escape

**Figure 1:**
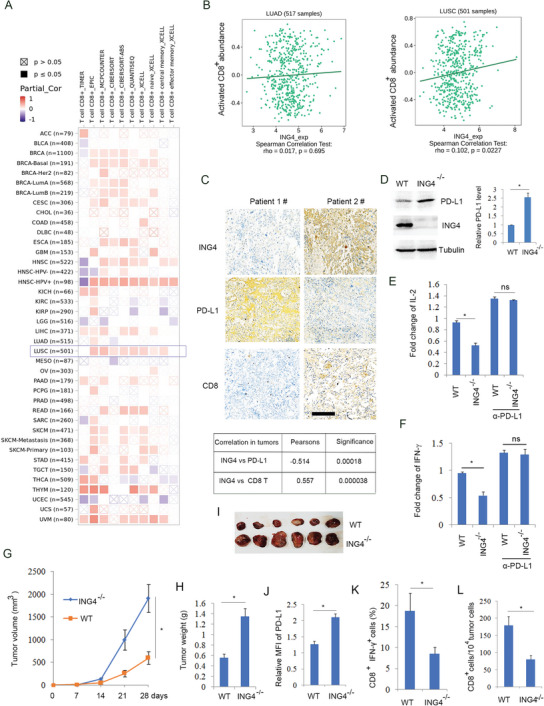
Loss of ING4 promoted tumor immune escape. A), The correlation of ING4 expression with T cell infiltration was assayed using TIME 2. http://timer.cistrome.org/. B), The correlation of ING4 expression with activated CD8^+^ T cell abundance was assayed using TISIDB (hku.hk). C), Immunohistochemical analysis of ING4, PD‐L1, and CD8 using LUSC tumor tissue specimens. Scale bar: 100 µm. The correlation of ING4 with PD‐L1, and CD8 protein expression was assayed (n = 48). D), Western blot analysis of wild type (WT) or ING4^−/‐^ H520 cell lysates. The relative PD‐L1 level was quantified. Results are expressed as means ± SEM (n = 3). *P<0.05. E,F) Co‐cultured Jurkat cells with cancer cells (WT or ING4^−/‐^ H520 cells) were treated without or with α‐PD‐L1 antibody (10 µg ml^−1^) for 24 h. The relative production of IL‐2 or IFN‐γ in supernatant was assayed. Results are expressed as means ± SEM (n = 3). *P<0.05. G‐I, WT or ING4^−/−^ LLC cells were inoculated subcutaneously into C57BL/6 mice. Tumor volume and weight were measured. Results are expressed as means ± SEM, n = 6. *P<0.05. J), Relative surface PD‐L1 level in WT or ING4^−/−^ LLC cell implanted tumors was assayed by flow cytometry. MFI: median fluorescence intensity. Results are expressed as means ± SEM (n = 6). *P<0.05. K), The percentage of CD8^+^/IFN‐γ^+^ T cells in WT or ING4^−/‐^ LLC cell implanted tumors was assayed by flow cytometry. Results are expressed as means ± SEM (n = 6). *P<0.05. L), The absolute number of CD8^+^ T cells in WT or ING4^−/‐^ LLC cell implanted tumors was assayed by flow cytometry. Results are expressed as means ± SEM (n = 6). *P<0.05.

Bioinformatic analysis shows that ING4 expression has a positive correlation with CD8^+^ T cell infiltration (**Figure** **1**A) and activated CD8^+^ T cells (Figure 1B) in lung squamous cell carcinoma (LUSC) but not in lung adenocarcinoma (LUAD). Since PD‐L1 on cancer cells binding to PD‐1 on T cells results in inhibition of T cell activity and proliferation,^[^ ^14^ ^]^ immunohistochemical analysis showed that high expression of ING4 was correlation a low level of PD‐L1 and a high level of CD8^+^ T cells in LUSC tissues (Figure 1C). These findings suggest that ING4 is a positive regulator of CD^+^ 8 T cells. To detect the effect of ING4 on PD‐L1 protein level, ING4 gene knockout H520 cells were developed by the CRISPR/Cas9 method. The results showed that ING4 knockout increased PD‐L1 protein level by Western blot analysis (Figure 1D; Figure S1A, Supporting Information), whereas it did not affect PD‐L1 gene expression (Figure S1B, Supporting Information). To further assay the effect of ING4 on T cell activity, co‐cultured Jurkat T cells with ING4 knockout H520 cells was performed. The results showed that ING4 knockout decreased Jurkat T cell IL‐2 and IFN‐γ production, while PD‐L1 antibody treatment rescued the effect (Figure 1E,F). Implanted tumor model analysis showed that ING4 knockout facilitated tumor growth (Figure 1G) and increased tumor weight (Figure 1H,I) but not in immunodeficient nude mice (Figure S2A–C, Supporting Information), which was agreement with increased PD‐L1 protein level (Figure 1J). Consistently, ING4 knockout markedly decreased cytotoxic T cell activity (Figure 1K) and CD8^+^ T cell numbers (Figure 1L), suggesting that loss of ING4 facilitated tumor immune escape by inhibiting T cell activity and proliferation, which was involved in increased PD‐L1 protein level.

### 2.2 ING4 Induced PD‐L1 Autophagic Degradation

**Figure 2:**
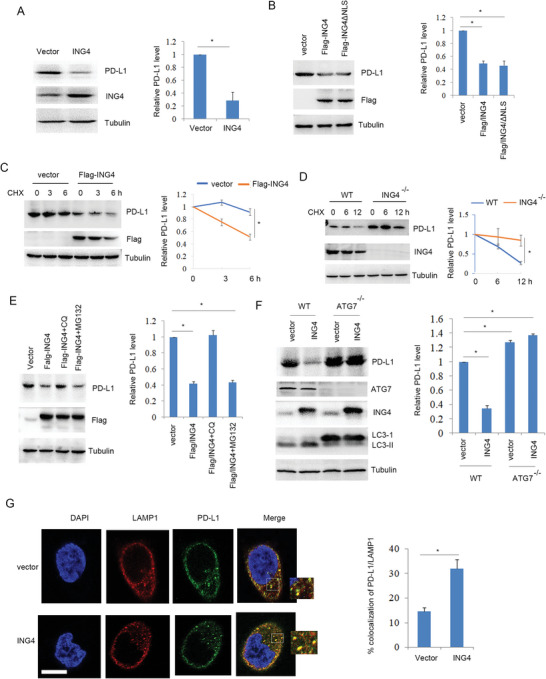
ING4 induced PD‐L1 autophagic degradation. A), H520 cells were transfected vector (pcDNA3) or Flag‐ING4 plasmids for 48 h and Western blot analysis of cell lysates. The relative PD‐L1 level was quantified. Results are expressed as means ± SEM (n = 3). *P<0.05. B, H520 cells were transfected vector (pcDNA3), Flag‐ING4 or Flag‐ING4/∆NLS plasmids for 48 h and Western blot analysis of cell lysates. The relative PD‐L1 level was quantified. Results are expressed as means ± SEM (n = 3). *P<0.05. C, H520 cells were transfected vector (pcDNA3) or Flag‐ING4 plasmids for 48 h. Cells were treated with cycloheximide (CHX, 30 µg ml^−1^) as indicated time course. Cell lysates were subjected to Western blot analysis. The relative PD‐L1 level was quantified. Results are expressed as means ± SEM (n = 3). *P<0.05. D, WT or ING4^−/‐^ H520 cells were treated with CHX (30 µg ml^−1^) as indicated time course. Cell lysates were subjected to Western blot analysis. The relative PD‐L1 level was quantified. Results are expressed as means ± SEM (n = 3). *P<0.05. E, H520 cells were transfected with vector (pcDNA3) or Flag‐ING4 plasmids for 48 h, and then cells were treated with CQ (30 µM), MG132 (20 µM) or DMSO as indicated for 4 h. Cell lysates were subjected to Western blot. The relative PD‐L1 level was quantified. Results are expressed as means ± SEM (n = 3), *P<0.05. F, WT or ATG7^−/‐^ H520 cells were transfected with vector (pcDNA3) or Flag‐ING4 plasmids as indicated for 48 h and Western blot analysis of cell lysates. The relative PD‐L1 level was quantified. Results are expressed as means ± SEM (n = 3). *P<0.05. G,H)520 cells were transfected with vector (pcDNA3) or Flag‐ING4 plasmids for 48 h, and the colocalization of LAMP1 with PD‐L1 was assayed by confocal. Scale bar: 25 µm. Percent colocalization of PD‐L1 with LAMP1 was quantified. Results are expressed as means ± SEM (n = 15 fields). *P<0.05.

Above results showed that ING4 knockout increased PD‐L1 protein level. In contrast, overexpression of ING4 significantly reduced PD‐L1 protein level (**Figure** **2**A; Figure S3A, Supporting Information) and it had no effect on its gene expression (Figure S3B, Supporting Information). ING4/ΔNLS (nuclear location signal) mutant inhibits its nuclear translocation^[^ ^2^, ^21^ ^]^ (Figure S4, Supporting Information). Further analysis showed that ING4/ΔNLS mutant reduced PD‐L1 protein level (Figure 2B), suggesting that cytoplasmic ING4 still reduced PD‐L1 protein level. Half‐life analysis showed that overexpression of ING4 significantly reduced PD‐L1 protein stability (Figure 2C). In contrast, loss of ING4 led to increased PD‐L1 protein half‐life (Figure 2D). These findings suggest that ING4 could induce PD‐L1 protein degradation. H520 cells treated with CQ (lysosome inhibitor) rather than MG132 (proteasome inhibitor) inhibited ING4‐mediated PD‐L1 degradation (Figure 2E), suggesting that ING4 promoted PD‐L1 degradation by autophagy. ING4‐induced PD‐L1 autophagic degradation was further detected using ATG7 gene knockout H520 cells. The results showed that deficiency of ATG7 decreased the accumulation of LC3B‐II and inhibited ING4‐mediated PD‐L1 autophagic degradation (Figure 2F). Confocal analysis showed that overexpression of ING4 facilitated the co‐localization of PD‐L1 with LAMP1 (lysosomal marker) (Figure 2G). These findings suggest that ING4 facilitated PD‐L1 autophagic degradation.

### 2.3 The Interaction of ING4 with PD‐L1

**Figure 3:**
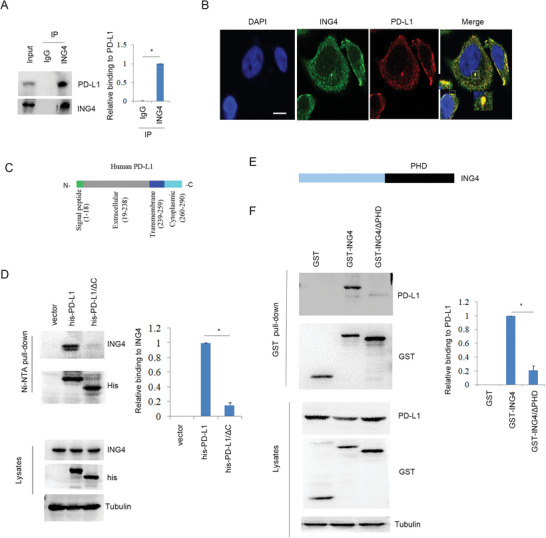
The interaction of ING4 with PD‐L1. A), Immunoprecipitation and Western blot analysis of H520 cell lysates. The relative binding of ING4 to PD‐L1 was quantified. Results are expressed as means ± SEM (n = 3). *P<0.05. B), The interaction of ING4 with PD‐L1 was assayed by confocal in H520 cells. Scale bar: 25 µm. C), Schematic illustration of PD‐L1. D), H520 cells were transfected vector (pcDNA3), his‐PD‐L1 or his‐PD‐L1/∆C plasmids as indicated for 48 h. Cell lysates were subjected to Ni‐NTA pull‐down and Western blot. The relative binding of his‐PD‐L1 to ING4 was quantified. Results are expressed as means ± SEM (n = 3). *P<0.05. E), Schematic illustration of ING4. PHD: plant homeodomain. F), H520 cells were transfected PEBG (GST), GST‐ING4, or GST‐ING4/∆PHD plasmids as indicated. Cell lysates were subjected to GST pull‐down and Western blot. The relative binding of GST‐ING4 to PD‐L1 was quantified. Results are expressed as means ± SEM (n = 3). *P<0.05.

Immunoprecipitation analysis showed that ING4 bound to PD‐L1 under basal condition (**Figure** **3**A; Figure S5, Supporting Information). In addition, ING4 was co‐localized with PD‐L1 in H520 cells by confocal analysis (Figure 3B). Ni‐NTA pull‐down analysis showed that ING4 bound to the cytoplasmic fragment of PD‐L1 (Figure 3C,D). GST pull‐down analysis showed that ING4 directly bound to PD‐L1, but the deleted PHD (plant homeodomain) of ING4 abolished this event (Figure 3E,F), suggesting that PHD was required for ING4 binding to PD‐L1. These findings suggest that ING4 could directly bind to PD‐L1.

### 2.4 The LIR Motif of ING4 was Required for Inhibition of Tumor Immune Escape

**Figure 4:**
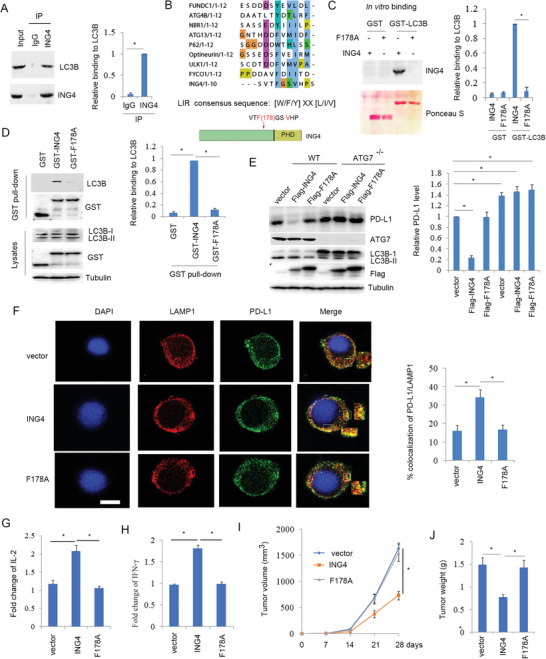
LIR motif of ING4 was required for PD‐L1 autophagic degradation and anti‐tumor immune escape. A), Immunoprecipitation and Western blot analysis of H520 cell lysates. The relative binding of ING4 to LC3B was quantified. Results are expressed as means ± SEM (n = 3). *P<0.01. B), Alignment of ING4 with LIR motif contained proteins, and schematic illustration of ING4 LIR motif. C), in vitro binding analysis of the interaction of ING4 with LC3B as described in methods. Down panel was ponceau S staining. The relative binding of ING4 to PD‐L1 was quantified. Results are expressed as means ± SEM (n = 3). *P<0.01. D, H520 cells were transfected PEBG (GST), GST‐ING4, GST‐F178A plasmids as indicated for 48 h. GST pull‐down analysis was performed using cell lysates. The relative binding of ING4 to LC3B was quantified. Results are expressed as means ± SEM (n = 3). *P<0.01. E, WT or ATG7^−/‐^ H520 cells were transfected vector (pcDNA3), Flag‐ING4 or Flag‐F178A plasmids for 48 h and Western blot analysis of cell lysates. The relative PD‐L1 level was quantified. Results are expressed as means ± SEM (n = 3). *P<0.01. F, H520 cells were transfected vector (pcDNA3), Flag‐ING4 or Flag‐F178A plasmids for 48 cells, and co‐localization of PD‐L1 with LAMP1 was detected by confocal. Scale bar: 25 µm. Percent colocalization of PD‐L1 with LAMP1 was quantified. Results are expressed as means ± SEM (n = 15 fields). *P<0.05. G,H, H520 cells were transfected vector (pcDNA3), Flag‐ING4 or Flag‐F178A plasmids for 48 h and co‐cultured with Jurkat cells for 24 h. IL‐2 or IFN‐γ release from Jurkat cells was assayed. Results are expressed as means ± SEM (n = 3). *P<0.05. I,J), Implanted tumor model analysis of vector (pLenti‐CMV), ING4 or F178A stably expressing LLC cells. Tumor volume and weight were measured. Results are expressed as means ± SEM, n = 6. *P<0.05.

In the autophagy process, LC3 plays an important role in the autophagosome development and targeted protein degradation in lysosome.^[^ ^22^, ^23^ ^]^ Above results demonstrated that ING4 mediated PD‐L1 autophagic degradation. For further determining the mechanism, immunoprecipitation analysis was performed. The results showed that ING4 bound to LC3B (**Figure** **4**A; Figure S6A, Supporting Information). Previous report suggests that a protein with LIR (LC3 interacting region) motif is required for LC3 binding and selective autophagy.^[^ ^22^ ^]^ Alignment analysis suggests that ING4 protein contains a LIR motif sequence (**F**178GS**V**) (Figure 4B). In vitro binding analysis showed that ING4 but not ING4/F178A mutant directly bound to LC3B (Figure 4C), which was accordant with the GST pull‐down analysis (Figure 4D). To further detect the effect of ING4‐LIR motif on PD‐L1 degradation, H520 cells were transfected with ING4 or F178A mutant plasmids. The results showed that ING4 reduced PD‐L1 protein level and half‐life, but ING4/F178A mutant abolished this event (Figure S6B,C, Supporting Information). In contrast, ATG7 gene knockout led to inhibition of autophagy and ING4‐mediated PD‐L1 degradation (Figure 4E). Immunofluorescent analysis showed that overexpression of ING4 facilitated the colocalization of PD‐L1 with lysosome, but F178A mutant abolished this event (Figure 4F). These findings suggest that ING4‐LIR motif plays an important role in regulating PD‐L1 degradation by autophagy. For further assessment of the effect of ING4‐LIR motif on T cell activity, co‐cultured Jurkat T cells with ING4 or F178A mutant expressing H520 cells were performed. The results showed that ING4 significantly increased T cell IL‐2 and IFN‐γ production, but F178A mutant did not (Figure 4G,H). Implanted tumor model analysis showed that ING4 but not F178A mutant significantly inhibited tumor growth and reduced tumor weight (Figure 4I,J), which was accordant with decreased PD‐L1 level (Figure S7A, Supporting Information) and increased cytotoxic T cell activity in tumors (Figure S7B, Supporting Information), suggesting that ING4‐LIR motif was required for inhibition of NSCLC immune escape, which was involved in reduced PD‐L1 level and increased T cell activity.

### 2.5 CK2 Induced ING4 Phosphorylation and Degradation

**Figure 5:**
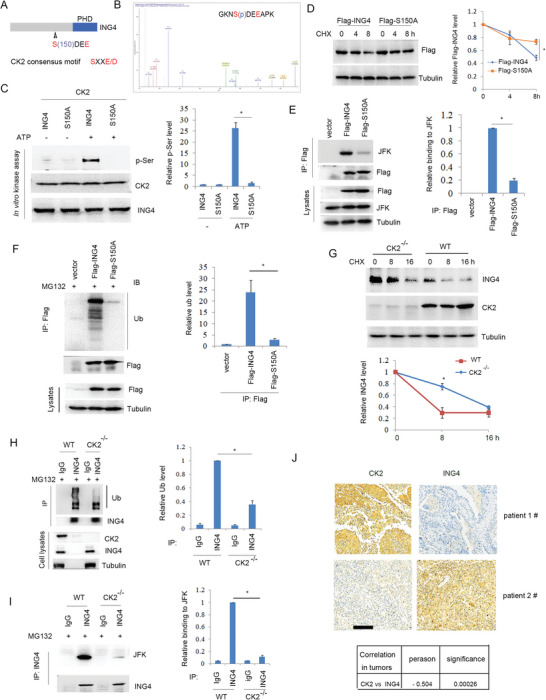
CK2 disabled ING4 protein by inducing its phosphorylation. A), Schematic illustration of the phosphorylated motif of ING4 by CK2. B), LC/MS/MS analysis of CK2‐induced ING4 phosphorylation as described in method. C), in vitro kinase assay of CK2‐induced ING4‐S150 phosphorylation as described in method. The relative p‐ser level was quantified. Results are expressed as means ± SEM (n = 3). *P<0.05. D, H520 cells were transfected pcDNA3‐Flag‐ING4 or pcDNA3‐Flag‐S150A plasmids for 48 h. Cells were treated with CHX (30 µg ml^−1^) as indicated time course. Cell lysates were subjected to Western blot. The relative Flag‐ING4 level was quantified. Results are expressed as means ± SEM (n = 3). *P<0.05. E, H520 cells were transfected vector (pcDNA3), Flag‐ING4 or Flag‐S150A plasmids for 48 h. Cell lysates were subjected to immunoprecipitation and Western blot. The relative binding of ING4 to JFK was quantified. Results are expressed as means ± SEM (n = 3). *P<0.05. F, H520 cells were transfected vector (pcDNA3), Flag‐ING4 or Flag‐S150A plasmids for 48 h, and cells were treated with MG132 (20 µM) for another 4 h to inhibit protein degradation. After that, immunoprecipitation and Western blot analysis were performed. The relative ubiquitination level was quantified. Results are expressed as means ± SEM (n = 3). *P<0.05. G, WT or CK2^−/‐^ H520 cells were treated with CHX (30 µg ml^‐1^) as indicated time course and Western blot analysis of cell lysates. The relative ING4 level was quantified. Results are expressed as means ± SEM (n = 3). *P<0.05. H, immunoprecipitation and Western blot analysis of WT or CK2^−/‐^ H520 cell lysates. The relative ubiquitination level was quantified. Results are expressed as means ± SEM (n = 3). *P<0.05. I, immunoprecipitation and Western blot analysis of WT or CK2^−/‐^ H520 cell lysates. The relative binding of ING4 to JFK was quantified. Results are expressed as means ± SEM (n = 3). *P<0.05. J, Immunohistochemical analysis of ING4 and CK2 using LUSC tumor tissue specimens. Scale bar: 100 µm. The correlation of CK2 with ING4 expression was assayed (n = 48).

ING4 could induce PD‐L1 autophagic degradation resulting in inhibition of NSCLC immune escape, while alignment analysis showed that ING4 contains a consensus CK2 phosphorylation motif (**Figure** **5**A), suggesting that CK2 could induce ING4‐S150 phosphorylation, which was demonstrated by LC/MS/MS (Figure 5B). In vitro phosphorylation analysis showed that CK2 induced ING4 phosphorylation, but S150A mutant reversed this event (Figure 5C). Half‐life analysis showed that ING4/S150A mutant increased ING4 protein stability (Figure 5D). Since JFK E3 ubiquitin ligase could induce ING4 ubiquitination and degradation,^[^ ^24^ ^]^ immunoprecipitation analysis showed that ING4 bound to JFK, but ING4/S150A mutant reduced this event (Figure 5E), which was consistent with ING4/S150A mutant suppressed ING4 ubiquitination (Figure 5F). To further assay the effect of CK2 on ING4 protein level, CK2 gene knockout H520 cells were developed by CRISPR/Cas9 method. The results showed that CK2 gene knockout increased ING4 protein stability (Figure 5G) and inhibited ING4 ubiquitination (Figure 5H), which was consistent with the reduced binding of ING4 to JFK ubiquitin ligase (Figure 5I). Clinical immunohistochemical analysis showed that CK2 protein levels were a negative correlation with ING4 in LUSC tissues (Figure 5J). These findings suggest that CK2 induced ING4‐S150 phosphorylation resulting in its ubiquitination and degradation.

### 2.6 ING4‐S150A Mutant Significantly Inhibited Tumor Immune Escape

**Figure 6:**
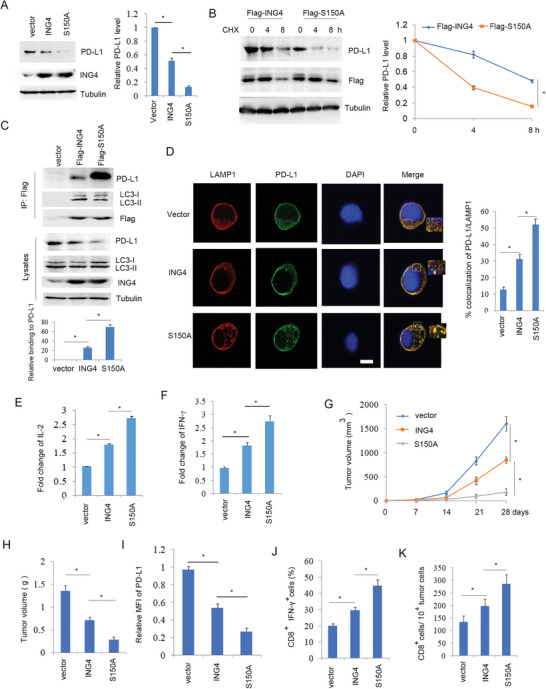
ING4/S150A mutant significantly increased PD‐L1 degradation and inhibited tumor immune escape. A), H520 cells were transfected vector (pcDNA3), Flag‐ING4, or Flag‐S150A plasmids for 48 h and Western blot analysis of cell lysates. The relative PD‐L1 level was quantified. Results are expressed as means ± SEM (n = 3). *P<0.05. B), H520 cells were transfected pcDNA3‐Flag‐ING4 or pcDNA3‐Flag‐S150A plasmids for 48 h. Cells were treated with CHX (30 µg ml^−1^) as indicated time course. Cell lysates were subjected to Western blot. The relative PD‐L1 protein level was quantified. Results are expressed as means ±SEM (n = 3). *P<0.05. C), H520 cells were transfected vector (pcDNA3), Flag‐ING4 or Flag‐S150A plasmids for 48 h. Cell lysates were subjected to immunoprecipitation and Western blot. The relative binding of ING4 to PD‐L1 was quantified. Results are expressed as means ± SEM (n = 3). *P<0.01. D, H520 cells were transfected vector (pcDNA3), Flag‐ING4 or Flag‐S150A plasmids for 48 h. The colocalization of PD‐L1 with LAMP1 was detected by confocal. Percent colocalization of PD‐L1 with LAMP1 was quantified. Results are expressed as means ± SEM (n = 15 fields). *P<0.05. E,F), H520 cells were transfected vector (pcDNA3), Flag‐ING4, or Flag‐S150A plasmids for 48 h and co‐cultured with Jurkat cells. IL‐2 or IFN‐γ release from Jurkat cells was assayed. Results are expressed as means ± SEM (n = 3). *P<0.05. G,H), Implanted tumor model analysis of vector (pLenti‐CMV), ING4 or S150A stably expressing LLC cells in C56BL/6 mice. I), Relative surface PD‐L1 expression in LLC cells stably expressing ING4 or the S150A mutant implanted tumors was assayed by flow cytometry. MFI: median fluorescence intensity. Results are expressed as means ± SEM (n = 6). *P<0.05. J), The percentage of CD8^+^/IFN‐γ^+^ T cells in LLC cells stably expressing ING4 or the S150A mutant implanted tumors was assayed by flow cytometry. Results are expressed as means ± SEM (n = 6). *P<0.05. K), The absolute number of CD8^+^ T cells in LLC cells stably expressing ING4 or the S150A mutant implanted tumor was assayed by flow cytometry. Results are expressed as means ± SEM (n = 6). *P<0.05.

Although CK2 induced ING4 phosphorylation and degradation, ING4‐S150A mutant reversed this event. Consistent with this, ING4‐S150A mutant significantly reduced PD‐L1 protein level and half‐life (**Figure** **6**A,B). Immunoprecipitation analysis showed that ING4/S150A mutant markedly increased the binding of ING4 to PD‐L1 (Figure 6C). Moreover, S150A mutant facilitated the co‐localization of PD‐L1 with LAMP1 (Figure 6D). These findings suggest that CK2/ING4 pathway maintained PD‐L1 protein stability by inducing ING4 degradation, which was reversed by ING4‐S150A mutant.

Co‐cultured analysis showed that ING4/S150A mutant significantly increased Jurkat T cell IL‐2 and IFN‐γ production (Figure 6E,F), suggesting that S150A mutant increased T cell activity. For further analysis of the effect of S150A mutant on NSCLC immune escape, implanted tumor model was performed using ING4 or S150A mutant stably expressed LLC cells. The results showed that ING4‐S150A mutant significantly decreased tumor growth (Figure 6G) and tumor weight (Figure 6H), which was in accordance with significantly reduced PD‐L1 protein level in tumors (Figure 6I). Furthermore, ING4‐S150A mutant markedly increased cytotoxic T cell activity and CD8^+^ T cell numbers compared to wild type ING4 (Figure 6J,K), suggesting that inhibition of ING4 phosphorylation significantly increased T cell activity and numbers resulting in blockade of NSCLC immune escape.

### 2.7 Loss of CK2 Inhibited NSCLC Immune Escape by Reducing PD‐L1 Level

**Figure 7:**
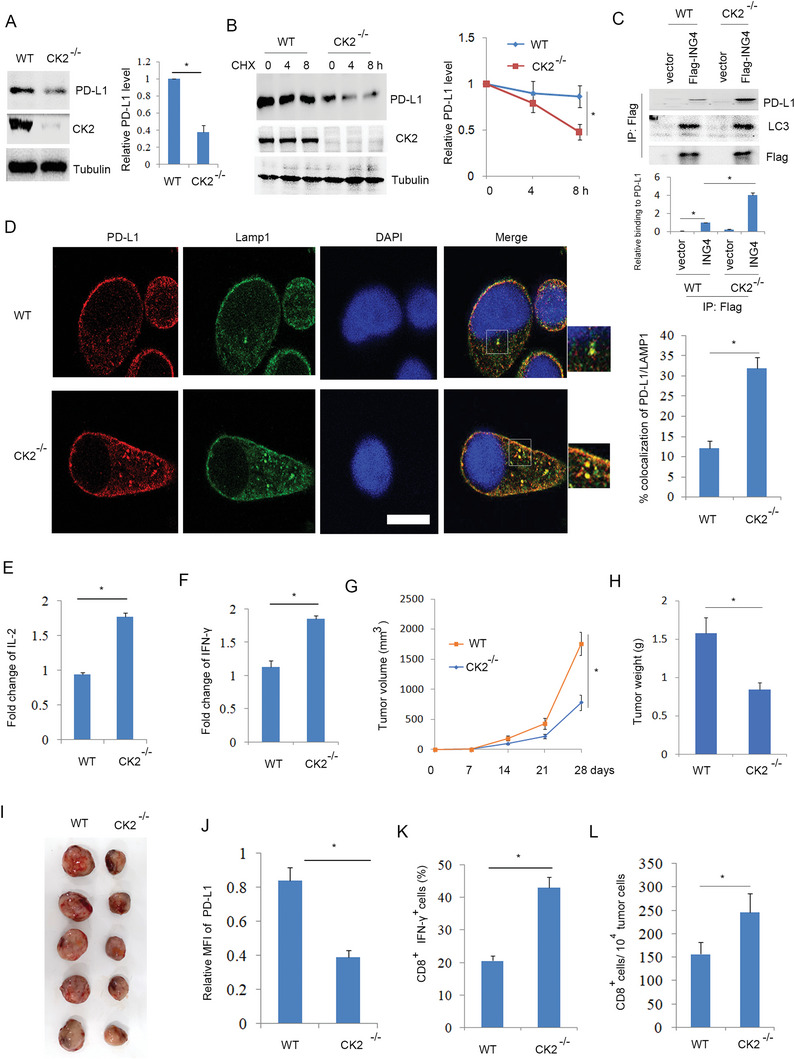
Loss of CK2 inhibited tumor immune escape. A), Western blot analysis of WT or CK2^−/‐^ H520 cell lysates. The relative PD‐L1 level was quantified. Results are expressed as means ± SEM (n = 3). *P<0.05. B), WT or CK2^−/‐^ H520 cells were treated with CHX (30 µg ml^−1^) as indicated time course and Western blot analysis of cell lysates. The relative PD‐L1 level was quantified. Results are expressed as means ± SEM (n = 3). *P<0.05. C), WT or CK2^−/‐^ H520 cells were transfected with vector (pcDNA3) or Flag‐ING4 plasmids for 48 h. Cell lysates were subjected to immunoprecipitation and Western blot. The relative binding of ING4 to PD‐L1 was quantified. Results are expressed as means ± SEM (n = 3). *P<0.01. D), Confocal analysis of WT or CK2^−/‐^ H520 cells. Scale bar: 25 µm. Percent colocalization of PD‐L1 with LAMP1 was quantified. Results are expressed as means ± SEM (n = 15 fields). *P<0.05. E,F), WT or CK2^−/‐^ H520 cells were co‐cultured with Jurkat cells. IL‐2 or IFN‐γ release from Jurkat cells was assayed. Results are expressed as means ± SEM (n = 3). *P<0.05. G–I), Implanted tumor model analysis of WT or CK2^−/−^ LLC cells in C57BL/6 mice. Results are expressed as means ± SEM (n = 5).*P<0.05. J), Relative surface PD‐L1 expression in WT or CK2^−/‐^ LLC cell implanted tumors was assayed by flow cytometry. MFI: median fluorescence intensity. Results are expressed as means ± SEM (n = 5). *P<0.05. K), The percentage of CD8^+^ /IFN‐γ^+^ T cells in WT or CK2^−/‐^ LLC cell implanted tumors was assayed by flow cytometry. Results are expressed as means ± SEM (n = 5). *P<0.05. L), The absolute number of CD8^+^ T cells in WT or CK2^−/‐^ LLC cell implanted tumors was assayed by flow cytometry. Results are expressed as means ± SEM (n = 5). *P<0.05.

Further analysis showed that CK2 knockout reduced PD‐L1 protein level and half‐life (**Figure** **7**A,B). In the CK2 knockout cells, ING4 enhanced the binding of PD‐L1 to LC3 by immunoprecipitation analysis (Figure 7C), and promoted the co‐localization of PD‐L1 with LAMP1 by confocal analysis (Figure 7D). These findings suggest that loss of CK2 facilitated ING4‐mediated PD‐L1 autophagic degradation. Co‐cultured analysis showed that deficient CK2 increased Jurkat T cell IL‐2 and IFN‐γ production (Figure 7E,F), suggesting that CK2 knockout enhanced T cell activity. Implanted tumor model analysis showed deficiency of CK2 significantly decreased tumor growth (Figure 7G) and tumor weight (Figure 7H,I) but not in immunodeficient nude mice (Figure S8A–C, Supporting Information), which was agreement with reduced PD‐L1 protein level in tumors (Figure 7J). Consistently, CK2 knockout increased cytotoxic T cell activity and CD8^+^ T cell numbers compared to wild type (Figure 7K,L). These findings suggest that loss of CK2 inhibited NSCLC immune escape by increasing T cell activity and numbers, which was involved in reduced PD‐L1 protein level.

### 2.8 Inhibitor of CK2 Enhanced NSCLC Immunotherapy

**Figure 8:**
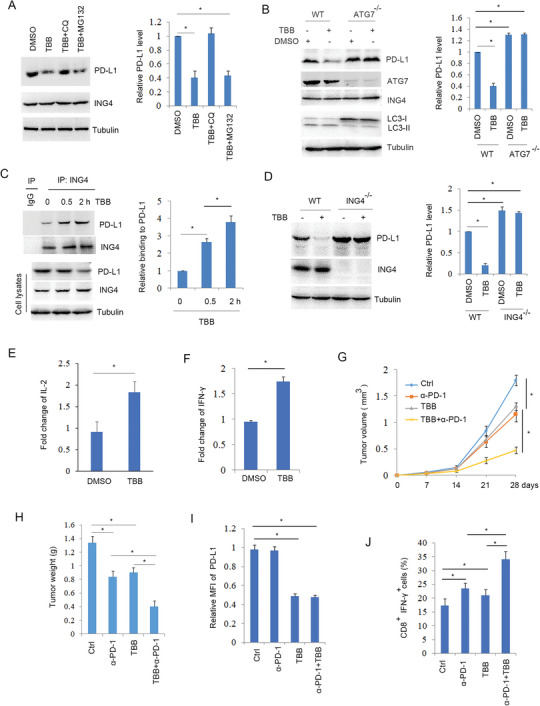
Inhibitor of CK2 enhanced antitumor immunotherapy. A), H520 cells were treated without or with TBB (10 µM), TBB (10 µM)+ CQ (30 µM), or TBB (10 µM)+ MG132 (20 µM) for 4 h and Western blot analysis of cell lysates. PD‐L1 level was quantified. Results are expressed as means ± SEM (n = 3). *P<0.05. B), WT or ATG7^−/‐^ H520 cells were treated without or with TBB (10 µM) for 4 h and Western blot analysis of cell lysates. PD‐L1 level was quantified. Results are expressed as means ± SEM (n = 3). *P<0.05. C), H520 cells were treated with TBB (10 µM) for 0, 0.5, and 2 h. Cell lysates were subjected to immunoprecipitation and Western blot. The relative binding of ING4 to PD‐L1 was quantified. Results are expressed as means ± SEM (n = 3). *P<0.05. D), WT or ING4^−/‐^ H520 cells were treated without or with TBB (10 µM) for 4 h and Western blot analysis of cell lysates. PD‐L1 level was quantified. Results are expressed as means ± SEM (n = 3). *P<0.05. E,F), co‐cultured Jurkat cells with H520 cells were treated with TBB (10 µM) for 24 h. IL‐2 or IFN‐γ production from Jurkat cells was detected. Results are expressed as means ± SEM (n = 3). *P<0.05. G,H), LLC cells were inoculated subcutaneously C57BL/6 mice. Mice were treated control, TBB, anti‐PD‐1 mice monoclonal antibody, or TBB+anti‐PD‐1 antibody as described in method. Tumor volume and weight were detected. Results are expressed as means±SEM, n = 5. *P<0.05. I), Relative surface PD‐L1 expression in LLC cell implanted tumors was assayed by flow cytometry. MFI: median fluorescence intensity. Results are expressed as means ± SEM (n = 5). *P<0.05. J), The percentage of CD8^+^ /IFN‐γ^+^ T cells in LLC cell implanted tumors was assayed by flow cytometry. Results are expressed as means ± SEM (n = 5). *P<0.05.

Despite the fact that CK2 induced ING4 phosphorylation and degradation, the CK2 inhibitor TBB decreased PD‐L1 levels (Figure S9A, Supporting Information). In addition, TBB treatment decreased PD‐L1 half‐life (Figure S9B, Supporting Information). These findings suggest that CK2 inhibitor TBB promoted PD‐L1 degradation. Further analysis showed that H520 cells treated with CQ (lysosome inhibitor) reversed the inhibition of TBB on PD‐L1 degradation (**Figure** **8**A). Blockade of autophagy using ATG7 gene knockout cells inhibited PD‐L1 degradation in response to TBB (Figure 8B). Immunoprecipitation analysis showed that TBB increased the binding of ING4 to PD‐L1 (Figure 8C). In ING4 gene knockout cells, TBB did not reduced PD‐L1 protein level (Figure 8D). These findings suggest that CK2 inhibitor TBB induced PD‐L1 autophagic degradation in an ING4 dependent manner.

To further assay the effect of TBB on T cell activity, H520 cells were treated with or without TBB and co‐cultured with Jurkat T cells. The results showed that TBB significantly increased Jurkat T cell IL‐2 and IFN‐γ production (Figure 8E,F), suggesting that TBB increased T cell activity, which was associated with reduced PD‐L1 protein level. To further analyze the effect of TBB on tumor immune escape, C57BL/6 mice were inoculated with LLC cells and treated with TBB or TBB together with PD‐1 antibody. The results showed that TBB inhibited tumor growth, but TBB together with PD‐1 antibody significantly enhanced this event (Figure 8G,H), which was in accordance with reduced PD‐L1 level in tumors (Figure 8I) and increased CD8^+^ T cell activity (Figure 8J). These findings suggest that CK2 inhibitor TBB effectively enhanced antitumor immunotherapy by inducing PD‐L1 degradation and increasing T cell activity.

## 3 Discussion

In cancer cells, PD‐L1 could be degradation by proteasomal or lysosomal‐dependent pathways.^[^ ^14^ ^]^ Induction of PD‐L1 degradation in proteasome is triggered by E3 ubiquitin ligases such as Cullin3,^SPOP[^ ^25^ ^]^ STUB1,^[^ ^26^ ^]^ β‐TrCP,^[^ ^27^ ^]^ RNF125,^[^ ^28^ ^]^ ITCH,^[^ ^29^ ^]^ ariadne RBR E3 ubiquitin protein ligase 1 (ARIH1),^[^ ^30^ ^]^ membrane‐associated RING‐CH 8 (MARCH8),^[^ ^31^ ^]^ and HRD1.^[^ ^32^ ^]^ Furthermore, PD‐L1 degradation in proteasome could be caused by albendazole/ubiquilin 4 pathway,^[^ ^33^ ^]^ annexin A1 ‐derived peptide A11/USP7 pathway,^[^ ^34^ ^]^ and ISG15 ubiquitin‐like protein.^[^ ^35^ ^]^ In addition to proteasomal‐dependent degradation, PD‐L1 could be degradation in lysosome by different pathways such as PKCα/GSK3β/MITF,^[^ ^36^, ^37^ ^]^ SA‐49/PKCα/GSK3β/MITF,^[^ ^38^ ^]^ IPAG/Sigma‐1,^[^ ^39^ ^]^ MTSS1/AIP4,^[^ ^40^ ^]^ TBM‐1/mTORC1/TFEB,^[^ ^41^ ^]^ and amlodipine/calcium flux.^[^ ^42^ ^]^ Moreover, HIP1R^[^ ^37^ ^]^ or PPARγ^[^ ^43^ ^]^ could directly bind to PD‐L1 and induce it autophagic degradation. As a tumor suppressor, ING4 plays an important role in regulating cancer cell proliferation, angiogenesis, DNA repair, apoptosis, migration, invasion, and chromatin remodeling.^[^ ^44^ ^]^ Our previous investigation suggests that ING4 could induce substrate ubiquitination and degradation,^[^ ^2^ ^]^ while lysosome inhibitor rather than a proteasome inhibitor terminated the effect of ING4‐mediated PD‐L1 degradation, suggesting that ING4 induced PD‐L1 autophagic degradation. Since LC3‐interacting region (LIR) motif containing proteins could act as a selective autophagy receptor for targeted protein lysosomal degradation.^[^ ^22^ ^]^ ING4 contains a LIR motif (T**F**(178)GS**V**), which was required for LC3 binding and mediated PD‐L1 autophagic degradation. Therefore, this study revealed that ING4 acts as an autophagy receptor to mediate PD‐L1 degradation in lysosome.

Although blockade of the PD‐1/PD‐L1 immune checkpoint could provide an effective immunotherapy for multiple types of cancer by activating the immune system to kill cancer cells, it has limited by the low response rates (10‐40%) for patients.^[^ ^14^, ^45^ ^]^ Cancer cells exhibit the ability to escape from immune surveillance by triggering multiple pathways such as c‐Myc, BRD4, HRE, and STAT3, which in turn increase PD‐L1 expression.^[^ ^13^ ^]^ PD‐L1 on cancer cells binds to PD‐1 on T cells leading to inhibition of T cell activity and proliferation resulting in tumor immune escape.^[^ ^12^ ^]^ Therefore, PD‐L1 degradation in cancer cells could effectively enhance anti‐tumor immunotherapy.^[^ ^14^ ^]^ In this study, we found that ING4 increased cytotoxic CD8^+^ T cell activity by inducing PD‐L1 degradation, consequently, inhibited NSCLC immune escape, and LIR motif of ING4 was required for this event. ING4 is an important tumor suppressor,^[^ ^44^ ^]^ which could bind to H3K4me3 and induce cell apoptosis in response to genotoxic stress.^[^ ^46^ ^]^ In addition, ING4 inhibits NFκB‐mediated pro‐inflammatory response,^[^ ^47^ ^]^ angiogenesis and tumor growth.^[^ ^48^ ^]^ However, it is unclear the effect of ING4 on tumor immune escape. This study revealed the inhibitory mechanism of ING4 on NSCLC immune escape.

Although ING4 could suppress tumor immune escape by inducing PD‐L1 autophagic degradation, loss of ING4 protein levels were observed in multiple types of cancer such as lung cancer,^[^ ^3^ ^]^ hepatocellular carcinoma (HCC),^[^ ^4^ ^]^ astrocytomas,^[^ ^5^ ^]^ ovarian,^[^ ^6^ ^]^ coloncancer,^[^ ^7^ ^]^ breast cancer,^[^ ^8^, ^49^ ^]^ and primary prostate tumors.^[^ ^50^ ^]^ However, the mechanism of loss of ING4 protein level in tumors is still unclear. Here we found that ING4 contains a CK2 phosphorylation motif, which was required for CK2‐mediated ING4/S150 phosphorylation, leading to recruitment of JFK ligase to induce ING4 ubiquitination and degradation. CK2 is a constitutively active serine/threonine kinase, which can trigger hundreds of substrates phosphorylation including Stat3, JAK2, p53, PTEN, RelA/p65, and AKT,^[^ ^18^, ^19^ ^]^ consequently, regulates multiple downstream signaling pathways that are involved in diseases development such as diabetes, cardiovascular diseases, angiogenesis, and cancer progression.^[^ ^20^ ^]^ CK2 regulates tumor progression because it is highly expressed in many types of cancer.^[^ ^19^ ^]^ However, the effect of CK2 on tumor immune escape is still unclear. In this study, we found that loss of CK2 reduced PD‐L1 protein level and increased T cell activity, which in turn inhibited tumor immune escape in LLC implanted tumor model. Moreover, ING4/S150A mutant significantly inhibited tumor immune escape compared to wild type ING4. This study revealed that CK2/ING4 pathway facilitated NSCLC immune escape. As blockade of PD‐1/PD‐L1 immune checkpoint could inhibit tumor immune escape,^[^ ^14^ ^]^ but it exhibits low response rates for patients.^[^ ^16^ ^]^ As one of the inhibitors of CK2, TBB induced PD‐L1 autophagic degradation, and combined TBB with PD‐1 mono‐antibody significantly inhibited tumor growth by increasing T cell activity in LLC implanted tumor model. This study could provide a potential therapy strategy for NSCLC immunotherapy.

Collectively, in NSCLC, CK2 induced ING4 ubiquitination and degradation, which in turn increased PD‐L1 protein level and inhibited T cell activity, consequently, promoted tumor immune escape. In contrast, CK2 inhibitor reversed this event, and combined CK2 inhibitor with PD‐1 antibody effectively enhanced tumor immunotherapy (Figure S10, Supporting Information).

## 4 Experimental Section

### Cells, Reagents, and Plasmids

H520, H1975, H1299, H226, HEK293T, LLC and Jurkat cells were obtained from the National Collection of Authenticated Cell Cultures of China. Jurkat cells were cultured in RPMI 1640 supplemented with 10% fetal bovine serum (FBS, Gibco). Other cells were cultured in DMEM supplemented with 10% FBS. Cells were mycoplasma negative using kit analysis (Vazyme). Human ING4 cDNA was cloned into PEBG. Human Flag‐ING4 cDNA was cloned into pcDNA3 vector. Human CSNK2A1 or his‐PD‐L1 cDNA was cloned into pcDNA3 vector. Mice ING4 cDNA was cloned into pLenti‐CMV vector. PEBG‐ING4/ΔPHD, Flag‐ING4/S150A, Flag‐ING4/F178A, or Flag‐ING4/ΔNLS, his‐PD‐L1/ΔC, pLenti‐ING4/F178A, or pLenti‐ING4/S150A were mutated by the site‐directed mutagenesis method. ATG7, ING4 or CSNK2A1 gene knockout cells were developed by CRISPR/Cas9 method, the plasmids were provided by YST BioTech (China). Protease inhibitor cocktail was obtained from Sigma. G418 sulfate, puromycin, and TBB were purchased from CSN Pharm (China).

### Antibodies, Immunoprecipitation, and Western Blot

ING4 (10617‐1‐AP), CK2α (10992‐1‐AP), Tubulin (11224‐1‐AP), Lamp1(21997‐1‐AP), PD‐L1(66248‐1), ATG7 (10088‐2‐AP), LC3B (14600‐1‐AP), GST (66001‐2), and Flag (66008‐4) antibodies were purchased from Proteintech. JFK (D127038) was purchased from Shangon Biotech. Cells were lysed in lysis buffer (50 mM TrisHCl pH7.4, 150 mM NaCl, 2 mM EDTA, 1% NP‐40, 0.1% SDS) with protease inhibitor cocktail. Proteins were subjected to SDS‐PAGE and transferred onto nitrocellulose membranes. Membranes were blocked in TBST with 5% non‐fat milk for 1 h, and then incubated membranes with primary antibodies overnight at 4 °C. After that, membranes were washed with TBST and incubated with secondary antibodies (Jackson Immunoresearch) for 1 h at room temperature. For immunoprecipitation, precleared lysates with protein A/G magnetic beads (Cat: B23202, Bimake) were added to primary antibodies and incubated overnight at 4 °C. After that, 12 µl protein A/G magnetic beads were added for another 4  h at 4 °C, and the beads were washed, the binding proteins were subjected to Western blot. Images were developed by chemiluminescence. Blots were quantified by Image J.

### Immunofluorescent Analysis

Cells were washed with PBS and fixed with 3.7% paraformaldehyde. First, cells were washed three times with PBS, permeabilized with 0.5% Triton‐X100 and washed, and blocked in 10% BSA for 1 h, and then incubated with primary antibodies as indicated, washed with PBS for three times, and incubated with secondary antibodies (Jackson Immunoresearch). Stained cells were viewed by a confocal microscope. Staining sections were quantified by Image J.

### In Vitro Binding Analysis

Human ING4 cDNA was cloned into pET28a vector. Human LC3B cDNA was cloned into PGEX‐6P. pET28a‐ING4/F178A was mutated by the site‐directed mutagenesis method. GST‐LC3B, his‐ING4, and his‐ING4/F178A were expressed in *E. coli* strain BL21. Protein was purified by Ni‐NTA beads or glutathione beads. For in vitro binding of ING4 to LC3B: GST‐LC3B(5 µg) fusion protein was immobilized on glutathione‐agarose beads in buffer (25 mM HEPES pH7.5, 6 mM NaCl, 0.2% NP‐40) for 1 h at 4 °C, and then the same amount of his‐ING4 or his‐ING4/F178A protein was added. These reactions were incubated for another 1 h. After that, beads were washed with PBS for three times, and the binding proteins were subjected to Western blot analysis.

### In Vitro Kinase Assay

Human ING4 cDNA was cloned into PGEX‐6P‐1 vector. GST‐ING4 and GST‐S150A mutant were expressed in *E. coli* strain BL21. The recombinant GST‐ING4 or GST‐S150A protein was purified by glutathione‐conjugated sepharose beads. Active CK2 protein was obtained from New England Biolabs (P6010S). 100 units active CK2, GST‐ING4 or GST‐S150A (10 ng) were added to the reaction buffer (50 mM Tris‐HCl, pH 7.5, 10 mM MgCl2 0.1 mM EDTA 2 mM DTT 0.01% Brij 35) for 30 min at 30 °C. The reactions were subjected to Western blot with indicated antibodies.

### Mass Spectrometry Assay

H520 cells were transfected with PEBG‐ING4 and CK2 plasmids for 48 h. GST pull‐down was performed, and samples were subjected to SDS‐PAGE. The gel‐purified ING4 proteins were digested by chyotrypsin and trypsin, and then the digested peptides were detected using UPLC‐Q‐Exactive (Thermo Fisher). Peptides coverage was 85% of ING4 amino acid sequence. The data were searched against UniProt database, peptide false discovery rate (FDR)<1%.

### Immunohistochemical Staining

Paraffin‐embedded lung squamous cell carcinoma (LUSC) tumor samples were obtained from Shanghai Wellbio Technology (China). Tissues were deparaffinized, rehydrated, and boiled in antigen retrieval buffer (pH 6.0, 10 mM citrate) for 30 min, and then washed, blocked, and stained with primary antibodies overnight at 4 °C. After that, sections were washed and incubated with secondary antibodies at room temperature for 1 h. Sections were stained by DAB (3,3′‐diaminobenzidine), and counterstained with hematoxylin.

### Jurkat Co‐Culture and ELISA Assay

Jurkat cells were activated using PMA (25 ng ml^−1^) and PHA (1 µg ml^−1^) for 24 h. Activated Jurkat cells were added to cancer cells at a ratio of 4:1 (Jurkat: cancer cells) for 24 h. The cell culture media were collected. An ELISA kit (MLBIO, China) was used to measure the amount of IL‐2 or IFN‐γ production by Jurkat cells.

### Mice Tumor Implantation and T Cell Activity Analysis in Tumors

WT or CK2^−/−^ LLC cells (1 × 10^5^), and WT or ING4^−/‐^ LLC cells (2 × 10^4^) were injected subcutaneously into 5‐weeks old male C57BL/6 mice. WT or CK2^−/−^ LLC cells (1 × 10^6^), and WT or ING4^−/‐^ LLC cells (1 × 10^6^) were injected subcutaneously into 5‐weeks old male nude mice. pLenti‐ING4, pLenti‐F178A, or pLenti‐S150A plasmids were transfected into HEK293T cells, and infected LLC cells using lentiviral particles. Stably expressing ING4, ING4/F178A, or ING4/S150A LLC cells (1 × 10^5^) were injected subcutaneously into 5‐weeks old C57BL/6 male mice. In other studies, LLC cells (1 × 10^5^) were injected subcutaneously into 5‐weeks old C57BL/6 male mice. Mice were treated with TBB (25 mg k^−1^g day^−1^), or TBB (25 mg k^−1^g day^−1^)+PD‐1 antibody (200 µg per mice^−1^, InVivoMAb anti‐mouse PD‐1, BioXcel). Tumor volume was measured with a digital caliper. Tumor volume = 1/2 (length × width^2^). All mice were obtained from Animal Center of Jiangsu University. All studies were carried out with the approval of the Jiangsu University Animal Care Committee (20 180 053).

Mice were sacrificed and tumors were collected. Tumors were cut into pieces and digested in DMEM with collagenase (2 mg ml^−1^, Biosharp) and DNase (10 µg ml^−1^) for 1 h at 37 °C, and then centrifuged and filtered using a 70 µm strainer in DMEM. Collected cells were added to blood cell lysis buffer for 5 min to remove red cells. After that, cells were filtered using a 40 µm strainer in PBS with 2% BSA. To assay membrane PD‐L1 protein level or CD8 numbers of cancer cells, cells (1 × 10^6^) were stained with PD‐L1 antibody (APC conjugated, Proteintech), CD8 (APC conjugated, Proteintech), or the corresponding isotype IgG control (APC conjugated, Proteintech). To assay T cell activity in tumors, cells were fixed with 4% paraformaldehyde for 20 min, and then permeabilized with 0.1% saponin. After that, cells were washed and co‐stained with CD8 (PE conjugated, Proteintech) and IFN‐γ (APC conjugated, Biolgend), or corresponding isotype IgG control at room temperature for 30 min. Cells were washed three times with PBS and analyzed by flow cytometry (CytoFLEX).

### Quantitative Real Time PCR

Total RNA was isolated using RNeasy kit (Sangon Biotech) and assayed using Real‐Time PCR assay kit (Takara). Relative mRNA expression levels were normalized against β‐actin. Fold change over control was determined according to the ΔCt method. PD‐L1 primers: Forward: 5‐ATGGAGAGGAAGACCTGAAGGTTCA‐3, reverse: 5′‐GGGGCATTGACTTTCACAGTAATTC GC‐3′; β‐actin primers: Forward: 5′‐GGTGGGCATGGGTCAGAAGGAT‐3′, reverse: 5′‐CACACGC AGCTCATTGTAGAAGGT‐3′.

### Data Availability

Figure 1A,B was generated from publicly available databases from TISIDB (http://cis.hku.hk/TISIDB), TIME 2. http://timer.cistrome.org/.

### Statistical Analysis

Data were expressed as the mean ± SEM. Differences between the two dependent groups were evaluated with the paired student's t‐test. *P*<0.05 was accepted as being statistically significant.

## Conflict of Interest

The authors declare no conflict of interest.

## Supporting information

Supporting Information

## Data Availability

The data that support the findings of this study are available in the supplementary material of this article.
