# Supplementary material for: Inhibition of CK2/ING4 Pathway Facilitates Non‐Small Cell Lung Cancer Immunotherapy
Source: Adv Sci (Weinh). 2023 Oct 23;10(34):2304068. doi: 10.1002/advs.202304068 (PMC10700192; doi:10.1002/advs.202304068)
Supplement: Supplementary file 1 — Supporting Information [file ADVS-10-2304068-s001.pdf]

ADVANCED SCIENCE
Open Access

Supporting Information

for *Adv. Sci.*, DOI 10.1002/advs.202304068

Inhibition of CK2/ING4 Pathway Facilitates Non-Small Cell Lung Cancer Immunotherapy

Qian Gou, Huiqing Chen, Mingjun Chen, Juanjuan Shi, Jianhua Jin, Qian Liu* and Yongzhong Hou**

Supplemental Figures and legends

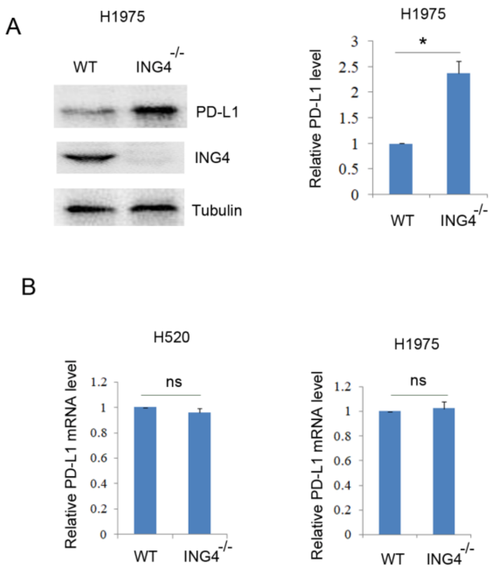

SFigure 1. ING4 reduced PD-L1 protein level without effect on its gene level.

A, Western blot analysis of WT or ING4$^{-/-}$ H1975 cell lysates. **B**, qPCR analysis of PD-L1 gene expression level in WT or ING4$^{-/-}$ H1975 cells. Results are expressed as means ± SEM, n=3. ns: no significance.

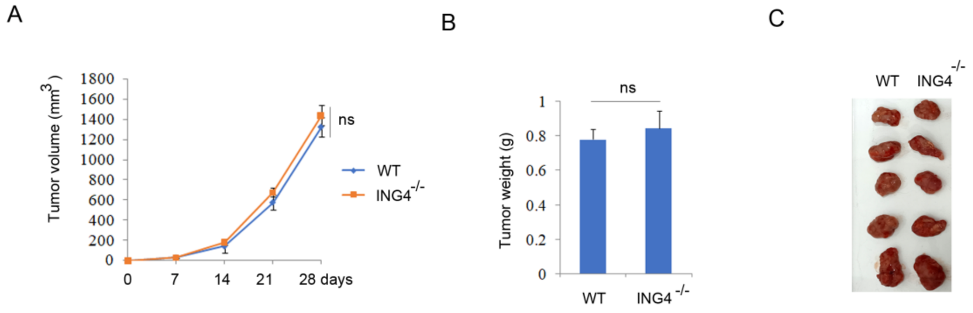

SFigure 2. Loss of ING4 had no significant effect on tumor growth in immunodeficient nude mice.

A-C WT or ING4$^{-/-}$ LLC cells were inoculated subcutaneously into nude mice. Tumor volume and

weight were measured. Results are expressed as means ± SEM, n=5. ns: no significance.

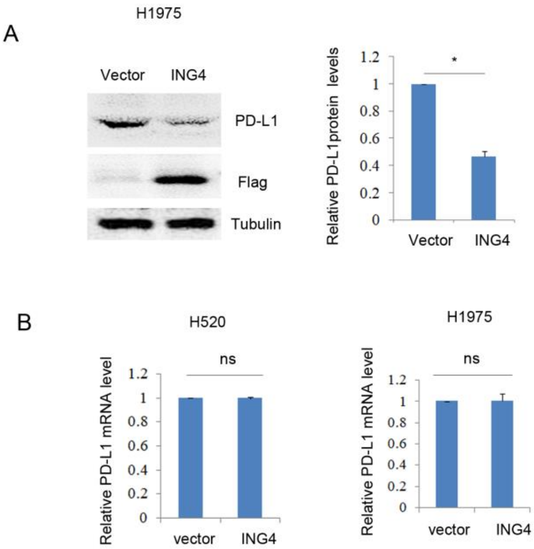

SFigure 3. Overexpression of ING4 reduced PD-L1 protein level.

A, H1975 cells were transfected vector (pcDNA3) or Flag-ING4 plasmids for 48h and Western blot analysis of cell lysates. PD-L1 protein level was quantified, Results are expressed as means ± SEM (n=3). **P*<0.05. **B**, H520 or H1975 cells were transfected vector (pcDNA3) or Flag-ING4 plasmids for 48h and qPCR analysis of PD-L1 gene expression level. Results are expressed as means ± SEM, n=3. ns: no significance.

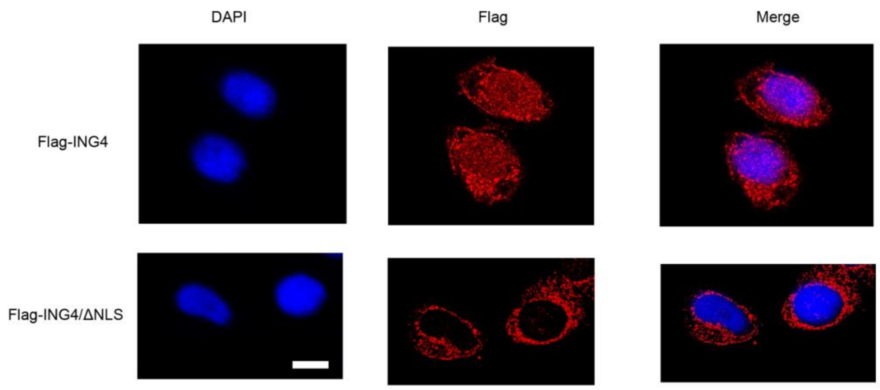

SFigure 4. ING4/ΔNLS (nuclear location signal) mutant inhibited its nuclear translocation.

H520 cells were transfected pcDNA3-Flag-ING4 or pcDNA3-Flag-ING4/ΔNLS plasmids for 48h, and then cells were subjected to confocal analysis. Scale bar: 25μm.

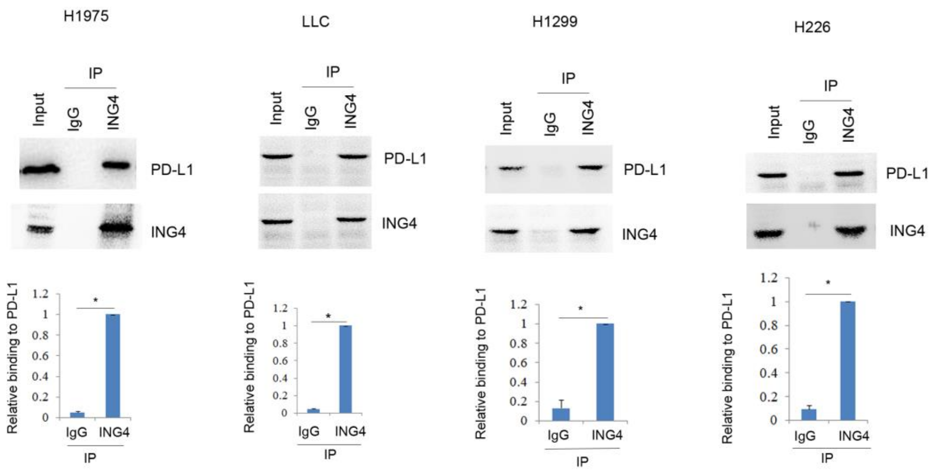

SFigure 5. The binding of ING4 to PD-L1.

H1975, LLC, H1299 or H226 cell lysates were subjected to immunoprecipitation and Western blot analysis. The relative binding of ING4 to PD-L1 was quantified. Results are expressed as means ± SEM (n=3). *P<0.05.

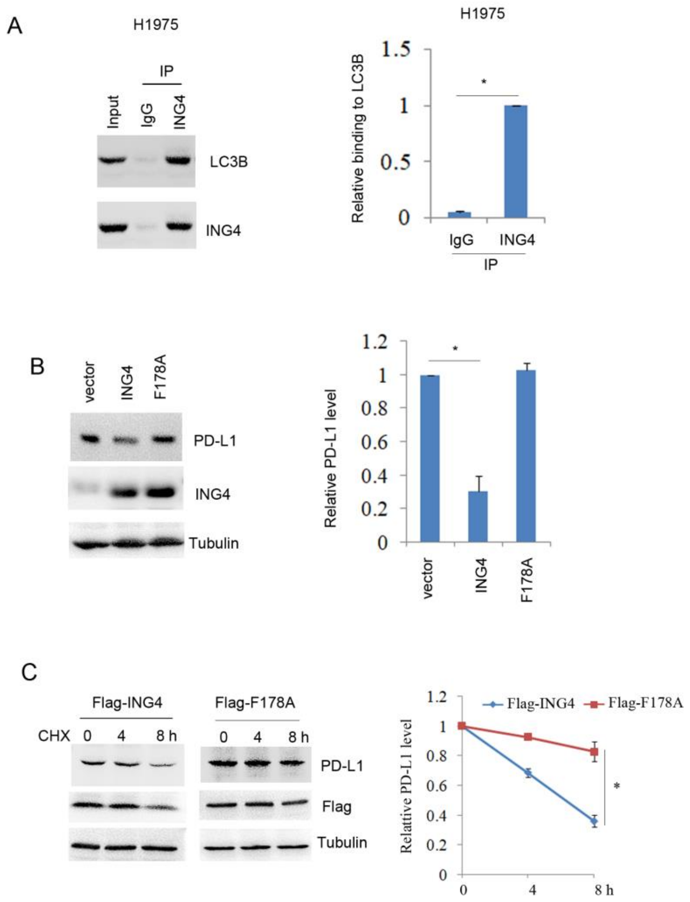

SFigure 6. LIR motif of ING4 was required for inducing PD-L1 autophagic degradation.

A, Immunoprecipitation and Western blot analysis of H1975 cell lysates. The relative binding of ING4 to LC3B was quantified. Results are expressed as means ± SEM (n=3). **P*<0.05. **B**, H520 cells were transfected vector (pcDNA3), Flag-ING4 or Flag-F178A plasmids for 48h and Western blot analysis of cell lysates. The relative PD-L1 level was quantified. Results are expressed as means ± SEM (n=3). **P*<0.01. **C**, H520 cells were transfected Flag-ING4 or Flag-F178A plasmids for 48h. Cells were treated with CHX (30 μg/ml) as indicated time course. Cell lysates were subjected to Western blot analysis. The relative PD-L1 protein level was quantified. Results are expressed as means ± SEM (n=3). **P*<0.05.

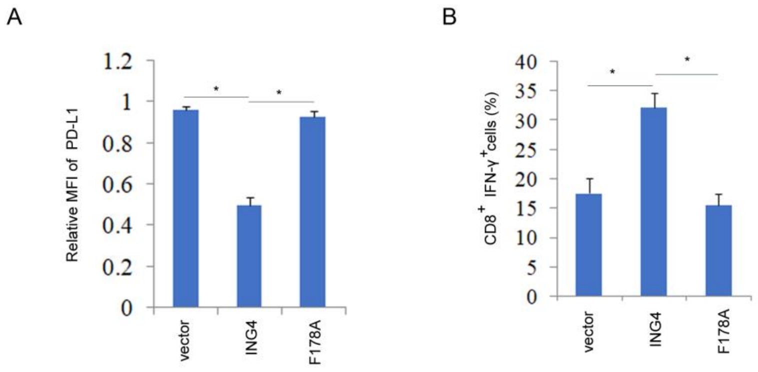

SFigure 7. ING4-LIR was required for activation of T cells by inducing PD-L1 degradation.

A, Relative surface PD-L1 expression in LLC cells stably expressing ING4 or F178A tumors was assayed by flow cytometry. MFI: median fluorescence intensity. Results are expressed as means ± SEM (n=6). **P*<0.05. **B,** The percentage of $CD8^+$/IFN-γ^+ T cells in LLC cells stably expressing ING4 or F178A tumors was assayed by flow cytometry. Results are expressed as means ± SEM (n=6). **P*<0.05.

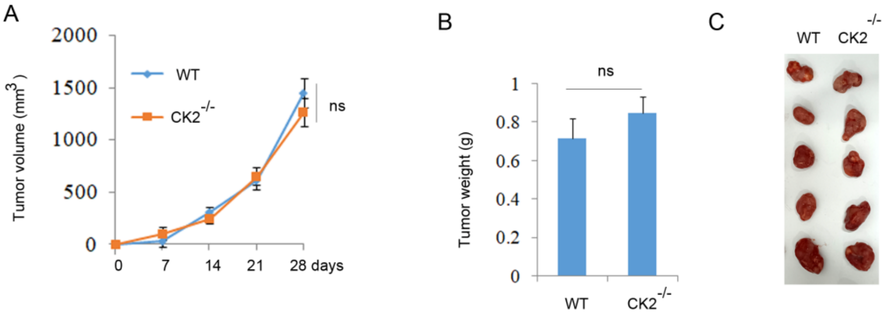

SFigure 8. Loss of CK2 had no significant effect on tumor growth in immunodeficient nude mice.

A-C, WT or $CK2^{-/-}$ LLC cells were inoculated subcutaneously into nude mice. Tumor volume and weight were measured. Results are expressed as means ± SEM, n=5. ns: no significance.

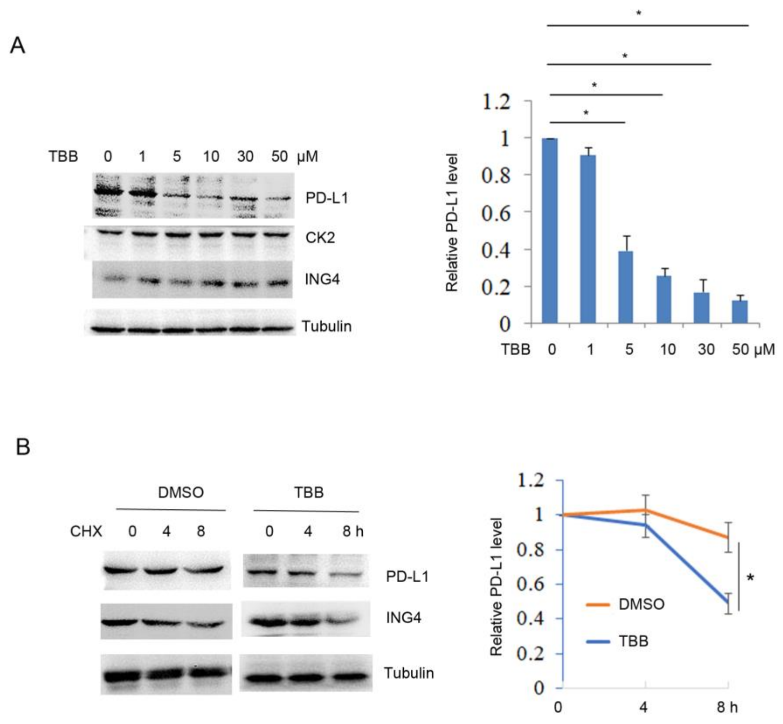

SFigure 9. CK2 inhibitor promoted PD-L1 degradation

A, H520 cells were treated with TBB as indicated dos course for 12h and Western blot analysis of cell lysates. The relative PD-L1 level was quantified. Results are expressed as means ± SEM (n=3). *P<0.05. **B**, H520 cells treated without or with TBB (10μM) together with CHX (30 μg/ml) as indicated time course and Western blot analysis of cell lysates. The relative PD-L1 protein level was quantified. Results are expressed as means ± SEM (n=3). *P<0.05.

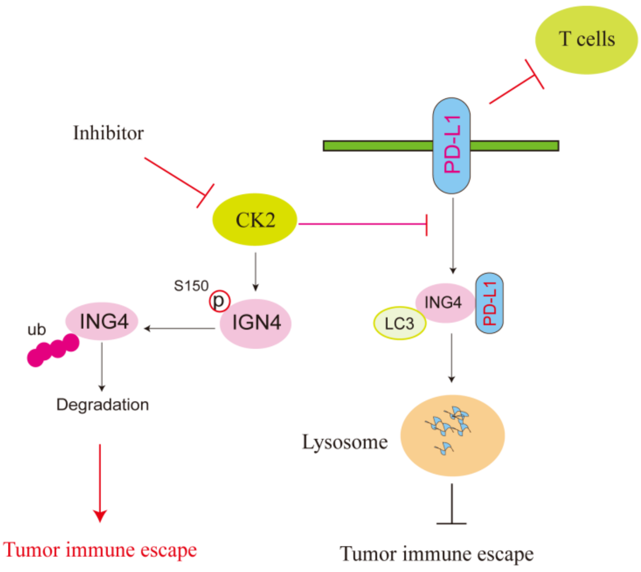

SFigure 10. Pathway of CK20-ING4 axis-mediated tumor immunotherapy.

ING4 acts as an autophagy receptor to induce PD-L1 autophagic degradation leading to inhibition of tumor immune escape, whereas CK2 could induce ING4-S150 phosphorylation and degradation by JFK ubiquitin ligase resulting in tumor immune escape. Importantly, CK2 inhibitor could effectively enhance PD-1 antibody anti-tumor immunotherapy by inhibiting CK2-ING4 pathway.
